# Scaling-up an efficacious school-based physical activity intervention: Study protocol for the ‘Internet-based Professional Learning to help teachers support Activity in Youth’ (*iPLAY*) cluster randomized controlled trial and scale-up implementation evaluation

**DOI:** 10.1186/s12889-016-3243-2

**Published:** 2016-08-24

**Authors:** Chris Lonsdale, Taren Sanders, Kristen E. Cohen, Philip Parker, Michael Noetel, Tim Hartwig, Diego Vasoncellos, Morwenna Kirwan, Philip Morgan, Jo Salmon, Marj Moodie, Heather McKay, Andrew Bennie, Ron Plotnikoff, Renata L. Cinelli, David Greene, Louisa R. Peralta, Dylan P. Cliff, Gregory S. Kolt, Jennifer M. Gore, Lan Gao, David R. Lubans

**Affiliations:** 1 Institute for Positive Psychology and Education, Australian Catholic University, Edward Clancy Building 167-169 Albert St, Strathfield, NSW 2135 Australia; 2 Priority Research Centre for Physical Activity and Nutrition, School of Education, University of Newcastle, Callaghan, NSW 2308 Australia; 3 Institute for Positive Psychology and Education and School of Exercise Science, Australian Catholic University, Edward Clancy Building 167-169 Albert St, Strathfield, NSW 2135 Australia; 4 School of Exercise Science, Australian Catholic University, Edward Clancy Building 167-169 Albert St, Strathfield, NSW 2135 Australia; 5 Physical Activity Research Group, School of Human Health and Social Sciences, Central Queensland University, Building 18, Yaamba Road, Rockhampton, QLD 4702 Australia; 6 Institute for Physical Activity and Nutrition (IPAN), School of Exercise and Nutrition Sciences, Deakin University, Geelong, Australia; 7 Deakin Health Economics, Centre for Population Health Research, Faculty of Health, Deakin University, Geelong, VIC Australia; 8 Center for Hip Health and Mobility, University of British Columbia, 7/F, 2635 Laurel Street, Vancouver, BC V5Z 1 M9 Canada; 9 School of Science and Health, Western Sydney University, Locked Bag 1797, Penrith, NSW 2751 Australia; 10 School of Education, Australian Catholic University, 250 Victoria Parade East, Melbourne, VIC 3002 Australia; 11 Faculty of Education and Social Work, University of Sydney, Sydney, NSW 2006 Australia; 12 Early Start Research Institute, School of Education, University of Wollongong, Wollongong, NSW 2522 Australia; 13 Teachers and Teaching Research Centre, School of Education, University of Newcastle, Callaghan, NSW 2308 Australia

**Keywords:** Cardiorespiratory fitness, Physical activity, Teacher professional development, Teacher professional learning, Online, Internet

## Abstract

**Background:**

Despite the health benefits of regular physical activity, most children are insufficiently active. Schools are ideally placed to promote physical activity; however, many do not provide children with sufficient in-school activity or ensure they have the skills and motivation to be active beyond the school setting. The aim of this project is to modify, scale up and evaluate the effectiveness of an intervention previously shown to be efficacious in improving children’s physical activity, fundamental movement skills and cardiorespiratory fitness. The ‘Internet-based Professional Learning to help teachers support Activity in Youth’ (*iPLAY*) study will focus largely on online delivery to enhance translational capacity.

**Methods/Design:**

The intervention will be implemented at school and teacher levels, and will include six components: (i) quality physical education and school sport, (ii) classroom movement breaks, (iii) physically active homework, (iv) active playgrounds, (v) community physical activity links and (vi) parent/caregiver engagement. Experienced physical education teachers will deliver professional learning workshops and follow-up, individualized mentoring to primary teachers (i.e., Kindergarten – Year 6). These activities will be supported by online learning and resources. Teachers will then deliver the *iPLAY* intervention components in their schools. We will evaluate *iPLAY* in two complementary studies in primary schools across New South Wales (NSW), Australia. A cluster randomized controlled trial (RCT), involving a representative sample of 20 schools within NSW (1:1 allocation at the school level to intervention and attention control conditions), will assess effectiveness and cost-effectiveness at 12 and 24 months. Students’ cardiorespiratory fitness will be the primary outcome in this trial. Key secondary outcomes will include students’ moderate-to-vigorous physical activity (via accelerometers), fundamental movement skill proficiency, enjoyment of physical education and sport, cognitive control, performance on standardized tests of numeracy and literacy, and cost-effectiveness. A scale-up implementation study guided by the RE-AIM framework will evaluate the reach, effectiveness, adoption, implementation, and maintenance of the intervention when delivered in 160 primary schools in urban and regional areas of NSW.

**Discussion:**

This project will provide the evidence and a framework for government to guide physical activity promotion throughout NSW primary schools and a potential model for adoption in other states and countries.

**Trial registration:**

Australia and New Zealand Clinical Trials Registry (ACTRN12616000731493). Date of registration: June 3, 2016.

## Background

Physical inactivity is a global pandemic, with “far-reaching health, economic, environmental and social consequences” [1]. Among children, the health benefits of physical activity are extensive and include improved physical fitness and bone health as well as reduced risk of obesity and cardiometabolic precursors of diseases such as type II diabetes [2, 3]. Physical activity may also improve psychological well-being and prevent mental health disorders such as depression and anxiety [3–5]. Recent evidence also indicates that, compared with their less active peers, physically active children can exert better cognitive control [6], are more engaged with school [7], and perform better on standardized tests of academic achievement [8].

The International Society for Physical Activity and Health [9] considers schools to be among the seven “best investments” for evidence-based physical activity promotion. Unfortunately, many schools are failing to provide children with sufficient opportunities to be active at school and do not equip them with the necessary skills and motivation to be active beyond the school setting [10, 11]. In systematic reviews, multi-component, flexible models were deemed more effective than single component models [12, 13]. Similarly, the US Centers for Disease Control and Prevention recommend schools deliver comprehensive school physical activity programs [14] that involve coordination across five components: (i) quality physical education (PE), (ii) activity during the school day, (iii) activity before and after school, (iv) staff involvement and (v) family and community involvement.

Despite convincing evidence of their effectiveness, few studies have implemented and evaluated comprehensive school physical activity programs. [15] Instead, most interventions have focused on one component of the school day (e.g., PE or recess/lunch breaks) [16, 17] and have neglected to address the multiple opportunities for physical activity promotion that exist within and beyond the school setting [18]. Among interventions that embraced a multi-component approach, few objectively measured effects on children’s physical activity (e.g., via accelerometers) [19].

The *SCORES* intervention was a comprehensive, multi-component physical activity and fundamental movement skills program for primary schools [20–22]. A socio-ecological model [23] provided the framework for the 12-month intervention, which consisted of components designed to engage teachers, students, parents and community sport organizations. Implementation strategies included: (i) professional learning and mentoring for teachers, (ii) feedback for teachers based on the quality of their PE and school sport, (iii) lesson resources for teachers, (iv) physical activity equipment, (v) school physical activity policy review and recommendations, (vi) training student leaders, (vii) parent engagement via newsletters, homework and information sessions, and (viii) engagement with local community sport. Our efficacy study [21] showed significant intervention effects at 12 months for cardiorespiratory fitness (5.4 laps; 95 % CI, 2.3 to 8.6), daily moderate-to-vigorous physical activity (12.7 mins/day; 5.0 to 20.5), and overall movement skill competency (4.9 units; -0.04 to 9.8). In addition, *SCORES* was delivered with a high degree of fidelity and teachers and students reported high satisfaction with the program.

There is a considerable gap between successful interventions like *SCORES*, and widespread dissemination in real world contexts [24, 25]. This is crucial, as to improve health of populations, interventions that have been effective in research settings must be delivered more broadly [26] and with less lag time between evidence generation and implementation. Indeed, there has been a proliferation of school-based physical activity intervention efficacy trials in recent years [18], yet these studies have made little impact on policy and practice [27].

In our project we will scale up and evaluate the effectiveness of a modified version of the *SCORES* intervention. The modified intervention centres around online delivery of professional learning to teachers. This customized, web-based delivery system was initially developed for a school-based physical activity intervention also led by our research team [28]. Teachers will deliver the intervention to students and parents and engage with community sport and recreation organizations. The modified intervention will be known as *iPLAY* (*I*nternet-based *P*rofessional *L*earning to help teachers to support *A*ctivity in *Y*outh) and will be among the first comprehensive school-based physical activity interventions with a large proportion of the program delivered online. A web-based delivery system is attractive as it may support scaling up and sustainability, and recent evidence indicates that online professional learning for teachers can be as effective as face-to-face training [29].

### Aims and hypotheses

We will conduct two complementary studies involving primary schools across New South Wales (NSW), Australia. In the first study, we will conduct a cluster randomized controlled trial (RCT) with a sample of 20 schools. The aim of this trial will be to evaluate the effectiveness and cost-effectiveness of *iPLAY* at 12 and 24 months, with cardiorespiratory fitness as the primary outcome. Key secondary outcomes will include objectively-measured moderate-to-vigorous physical activity, fundamental movement skills, cognitive control and student performance on standardized tests of numeracy and literacy. We hypothesize that:

1. compared with the control condition, the *iPLAY* intervention will produce positive effects on children’s outcomes in the short-term (post-intervention, 12 months after baseline, primary endpoint for the trial),
2. these benefits will be maintained 12 months after the end of the intervention (24 months after baseline), and
3. the intervention will represent value-for-money.

The aim of the second study will be to evaluate the intervention’s wide-scale implementation (scale up). To achieve this goal we will adopt the RE-AIM framework and assess Reach, Effectiveness, Adoption, Implementation, and Maintenance of *iPLAY* in 160 NSW primary schools (i.e., Kindergarten – Year 6).

## Methods

### Design

**Fig. 1:**
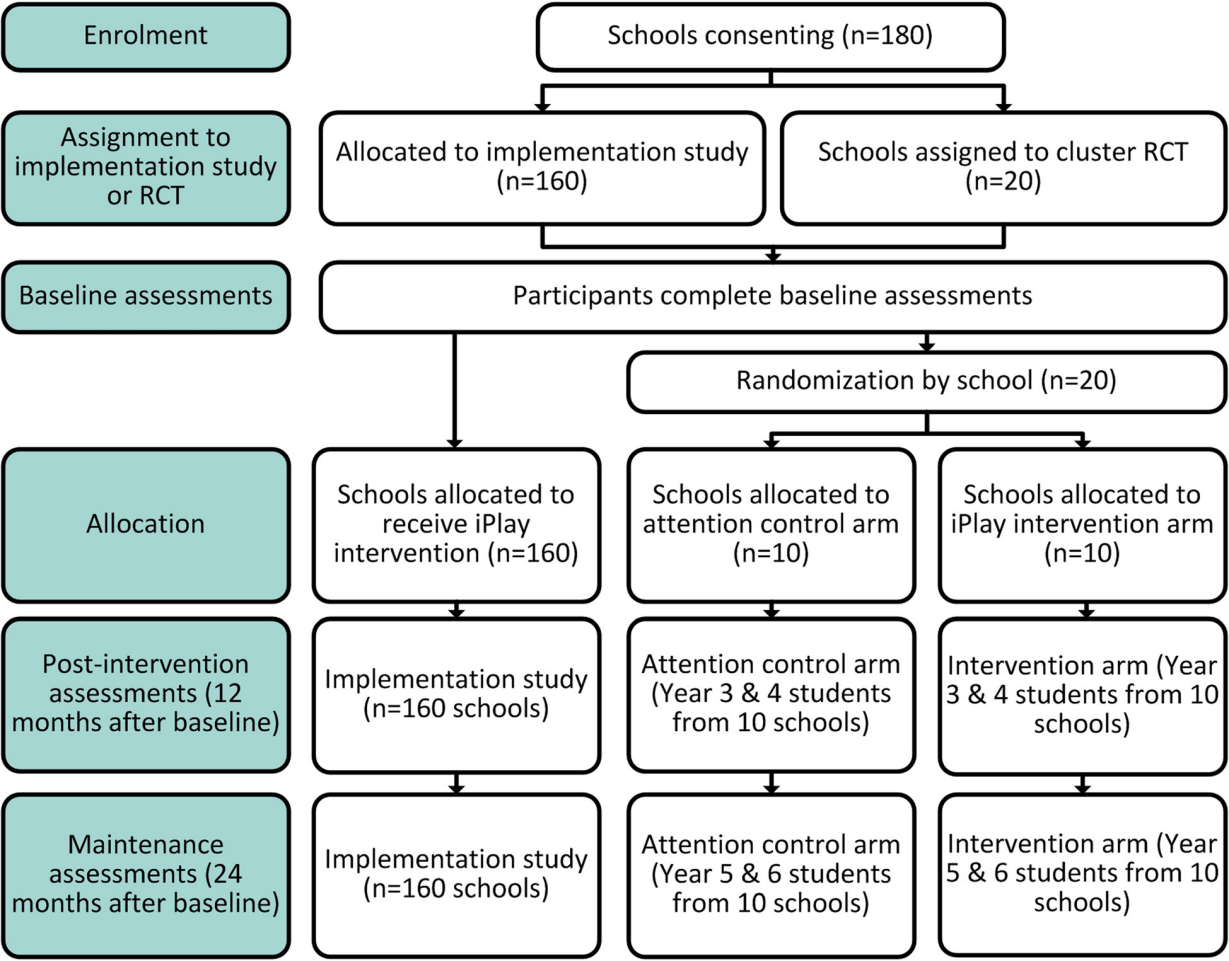
Modified CONSORT Flow Diagram

We will concurrently conduct two complementary evaluations (see Fig. 1):

1. A cluster RCT involving 20 schools (1:1 allocation to intervention and attention control conditions) to evaluate the *E*ffectiveness and incremental cost-effectiveness of the *iPLAY* intervention, with cardiorespiratory fitness as the primary outcome.
2. A scale-up implementation study will examine *iPLAY*’s *R*each, *A*doption, *I*mplementation and *M*aintenance with a reduced examination of *E*ffectiveness and cost-effectiveness. These aspects will be measured in 160 schools.

All teachers in each school selected for the cluster RCT and scale-up implementation study will be invited to complete the *iPLAY* intervention (or attention control intervention for 10 schools in the cluster RCT). However, only the student cohorts in Years 3 and 4 at baseline will complete outcome assessments (i.e., students in Years 3 and 4 at baseline, students in Years 4 and 5 at post-intervention [12 months], students in Years 5 and 6 at maintenance [24 months]). These students will be available for assessment at all time-points (c.f. most Year 5 and 6 students will leave the school by 24 months), and will have the cognitive ability to complete the questionnaires (c.f., Years 1 and 2). In addition, these years represent the ideal period to develop fundamental movement skill competency [30], which may help prevent the decline in physical activity typically observed during the transition from childhood to adolescence [31].

### Recruitment, selection and randomization for both investigations

All government-funded NSW primary schools (*N* = 1,600) [32] will be invited to participate in the project. All schools will be eligible to participate in the scale-up implementation study, but those designated as ‘Schools for Specific Purposes’ (i.e., for students who require intensive levels of support) will not be eligible for the cluster RCT. Schools that participated in the original *SCORES* efficacy study [21] will be eligible for the scale-up implementation study, but will be excluded from the cluster RCT.

Schools will be recruited via presentations at conferences and meetings (e.g., regional meetings of the NSW Primary Principals Association) and advertisements sent by the NSW Department of Education and the Australian Council for Health, Physical Education and Recreation. We will also advertise via the NSW Department of Education Twitter feeds and Facebook pages. We aim to recruit a total of 180 schools (>10 % of the total number of NSW government-funded primary schools). From the schools that express interest prior to May 2016, we will use a computer-generated algorithm to randomly select 90 to form Cohort 1. Recruitment will continue through to March 2017 at which point we will randomly select 90 schools to form Cohort 2.

From within each cohort, we will select 10 schools to participate in the cluster RCT; the other 80 schools will participate in the scale-up implementation study. Selecting schools for the cluster RCT will involve a four-step approach. The aims of this process are to ensure that schools in the cluster RCT are: (i) broadly representative of schools in NSW and (ii) assigned to trial arms such that most school-level covariates (e.g., socioeconomic status [SES], geographic location) are balanced, thereby increasing the likelihood that children in the two conditions are similar on the outcome variables at baseline. The four steps are:

1. *Stratification:* All schools that express interest in the study and are among the 90 selected to participate in each cohort will be stratified according to SES and geographic location. Given the number of schools is small, the stratification process will require relatively coarse groupings. The Index of Community Socio-educational Advantage (ICSEA) will serve as the SES variable. This index includes information regarding parental SES and Indigenous representation [33]. The index has a median of 1000 and ranges Australia-wide from 300 to 1300 indicating heavy negative skew [33]. NSW has a similar distribution ranging from 582 to 1202 with a median of 1003. We will split the sample into a higher SES stratum (ICSEA < =1003) and a lower SES stratum >1003. These strata will be further split by geographic distribution using the Australian Bureau of Statistics remoteness index by postcode. The index has 12 categories but this will be reduced to two: urban (less remote) and provincial (more remote). This process will produce four strata: (i) urban-higher SES, (ii) urban-lower SES, (iii) provincial-higher SES, and (iv) provincial-lower SES.
2. *Match-pairing:* We will employ a Euclidian distance minimization strategy to create pairs of similar schools from within strata. The variables used in this minimization process will be: (i) ICSEA, (ii) school size (number of students enrolled), (iii) average scores on national standardized test of numeracy and literacy that are completed by all NSW children (see outcomes section for further details) and (iv) school participation (or not) in a state-wide physical activity and nutrition program, known as Live Life Well at School [34], that took place from 2008 to 2015.
3. *Pair selection:* Once schools have been matched using the minimization procedure, we will select the two or three most similar pairs of schools from within each stratum to participate in the cluster RCT. Through this process, four schools will be selected from each of the provincial strata and six schools are chosen from each of the urban strata. This strategy will allow for calculation of average treatment effects and differences in treatment effects by strata.
4. *Randomization*: Following baseline data collection, schools will be randomly assigned from within each pair to the experimental or control arm of the cluster RCT. An experienced statistician who is not part of the research team will conduct the randomization procedure using a computer-generated algorithm.

From within each cohort of 90 schools, the 80 schools not selected for the cluster RCT will be included in the scale-up implementation study.

### Intervention

**Fig. 2:**
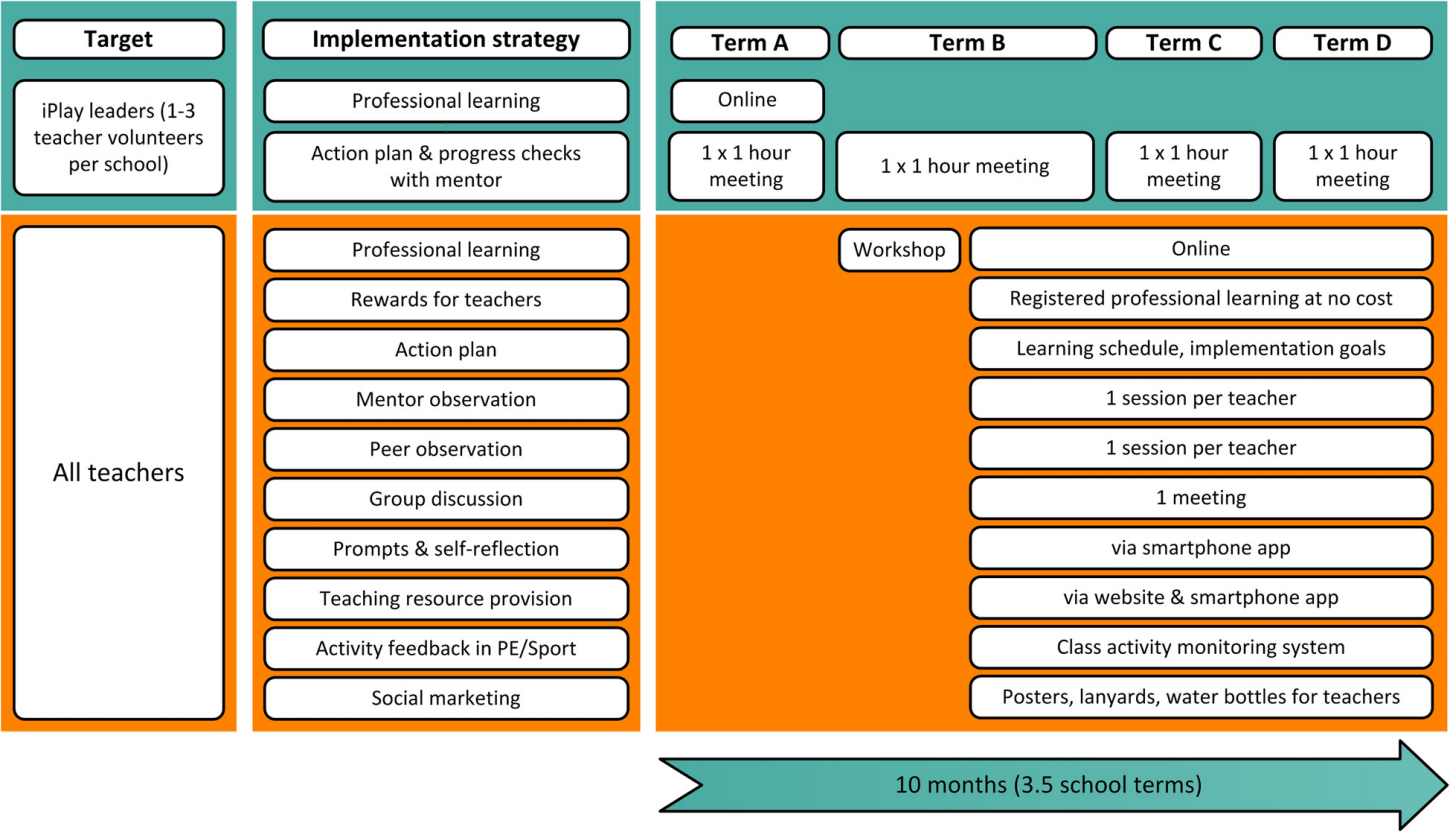
iPLAY Intervention Implementation Strategies

The intervention design and delivery will be identical for schools in the cluster RCT and the scale-up implementation study. *iPLAY* will include six components to promote physical activity participation and fundamental movement skill competency (see Table 1). An ‘*iPLAY* Mentor’ (employed by the project) will deliver a professional learning workshop and follow-up individualized mentoring to primary teachers. These activities will be supported by an online learning and resource platform (see implementation strategies in Fig. 2). Teachers within the schools will then deliver intervention components. All classroom teachers will deliver curricular components of the intervention (e.g., quality PE and school sport). Within each school the principal will identify up to three classroom teachers as ‘*iPLAY* Leaders’. Leaders will deliver non-curricular components of the intervention (e.g., active playgrounds) and support other teachers with implementation of curricular components.

**Table 1:** iPLAY Intervention Components

Curricular Components	Description	Implementation Measurement
Quality PE and school sport	• Teachers will deliver 150 minutes of planned PE or school sport each week. • Lessons will be delivered according to the SAAFE principles (Supportive, Active, Autonomous, Fair and Enjoyable). • Students will spend >40 % of PE/sport lesson time being physically active (i.e., in MVPA).	• Classroom teachers will self-report delivery of PE and School Sport on eight occasions during the intervention at the start of each online learning module. • Mentors will observe and rate each teacher’s delivery using the SAAFE checklist once during the intervention. • Monitored using the class activity tracking system provided to each school.
Classroom movement breaks	• Teachers will deliver 2 × 3-minute classroom energizer activities per day (30 minutes per week)	• Teachers will self-report at the start of each of the eight online learning modules. • Teachers usage of the video-based classroom movement breaks on the website (resources section) will be monitored.
Physically active homework	• Teachers will provide one physically active homework activity per week (except in schools that have a ‘no homework’ policy)	• Teachers will self-report at the start of each of the eight online learning modules.
Non-Curricular Components	Description	Implementation Measurement
Active playgrounds	• Children will spend >40 % of recess and lunch breaks in MVPA.	• Leaders will rate via the website their implementation of active playground strategies. Ratings will occur three times during the intervention (during meetings with mentors). • Student physical activity during breaks will be measured via accelerometry at each assessment time-point (baseline, 12 months, 24 months), but will not be measured during the intervention.
Community physical activity links	• Schools will utilize the ‘Sporting Schools’ funding to offer after-school physical activity program at least once per week across two school terms. • During the intervention at least one teacher in each school will complete accreditation/training procedures with a recognized sporting body that will allow them to deliver the Sporting Schools’ program in their school.	• Principals will report on all non-curricular sport and recreation in each school. • Teachers will report the sport accreditation/training they complete.
Parent and caregiver engagement	• Schools will deliver 1 × newsletter item per fortnight, which will include a link to the parent portion of the *iPLAY* website. • Schools will deliver 2 × *iPLAY* update presentations to parents per year during existing parent-teacher events. • Schools will organize one physically active school fundraising event each year.	• Leaders will record via the website the frequency of newsletter distribution and parent meetings. • Parent access to the *iPLAY* website will be monitored. • Leaders will provide evidence of school fundraiser events.

*Note*: *MVPA* moderate to vigorous physical activity

#### iPLAY mentors

Mentors will be current and recently retired teachers with NSW Board of Studies Teaching and Educational Standards (BOSTES) specialist accreditation in Health and PE. These specialist teachers are ideally placed to deliver *iPLAY* as primary school teachers will regard them as credible. In addition to holding BOSTES accreditation in Health and PE, inclusion criteria for mentors will include: (i) smartphone ownership, (ii) basic computer skills, (iii) a valid driver’s license and (iv) access to a vehicle to travel to schools. Mentors will be recruited via professional associations (Australian Council for Health Physical Education and Recreation), NSW Department of Education social media advertising and the project team’s existing professional networks.

The project will provide funding to schools to cover the cost of a substitute teacher when current teachers who become mentors are seconded to work on *iPLAY*. Current teachers will receive no direct payment, but their training and participation will earn them credit towards designation as a BOSTES ‘Highly Accomplished Teacher’. Achieving this level of accreditation increases teachers’ salaries and is required for those seeking school leadership roles (e.g., Principal).

The project will pay retired teachers a rate ($400/day or $200/half-day) that is equivalent to the rate for substitute teachers in NSW. All mentors will be reimbursed for travel expenses when travelling to schools more than 25 km from their home.

##### iPLAY mentor training

During two 7-h face-to-face workshops, the project team will train mentors to deliver the intervention. Workshops will include: (i) familiarization with the intervention components and procedures and their rationale, (ii) review of answers to predetermined ‘frequently asked questions’, (iii) discussion regarding methods to establish mentors’ credibility, ‘relatability’ and likeability [35], (iv) problem solving exercises regarding likely challenging scenarios, and (v) role-playing exercises.

##### iPLAY mentor delivery

As shown in in Fig. 2, mentors will complete the following tasks in each school:

1. Meet with *iPLAY* leaders to facilitate implementation of non-curricular intervention components (4 × 1 hour meetings – 1 per term). In most cases, these meetings will be conducted face-to-face on the same day as mentors visit schools to observe teachers’ delivery of PE and school sport lessons. However, in some circumstances (e.g., very small schools in which mentors only need to visit once or twice to observe all classroom teachers’ PE/school sport lessons), a teleconference or internet-mediated videoconference may be chosen to complete this meeting.
2. Deliver a 2-hour workshop at the school to all teachers. The workshop will focus on the curricular components of the intervention. It will include a 1-hour classroom session in which the mentors will present information videos with *iPLAY* content and then facilitate discussion and activities using presentation slides provided by the project. The workshop will also include a 1-hour practical session in which the mentor will demonstrate quality teaching using a lesson plan provided by the project.
3. Observe one PE or school sport lesson for each teacher and provide feedback to the teacher during a 30-minute meeting. This observation and feedback process will require mentors to visit each school, with the number of visits determined by the number of teachers in the school. On average, we expect mentors to visit once per term.

Methods to ensure high quality and consistent delivery of the workshop and the observation feedback meetings include:

1. At the end of the training workshops and before delivering the intervention in schools, mentors will complete an examination regarding project procedures and workshop content (e.g., answers to frequently asked questions).
2. During the face-to-face workshops, mentors will deliver all content to teachers using videos produced by the project team.
3. Discussion of video content and learning activities for teachers during the workshop will be based on slides and a lesson plan provided by the project team.
4. Mentors will access videos and presentation slides through the project website. Thus, the project team will be able to verify if and when each component was accessed.
5. The project team will provide mentors with answers to frequently asked questions for each workshop, and update this list as the project progresses.
6. Mentors will upload their lesson observations using a structured template within the project website or smartphone app (iOS and Android versions will be available).
7. Mentors will participate in bi-annual meetings that will provide them with ongoing professional learning and support. The project team will lead these face-to-face meetings.

#### Curricular components – classroom teachers

Classroom teachers will participate in professional learning designed to help them implement the curricular intervention components. This training will involve a 2-h workshop (face-to-face), 4 h of online learning (8 × 30 minute modules), a mentoring meeting, a peer observation, and a discussion at a staff meeting focused on *iPLAY* implementation. Completion of these activities will provide each classroom teacher with 14 h of professional learning that is registered with NSW BOSTES. To maintain their accreditation, NSW teachers are required to accumulate 50 h of BOSTES registered professional learning every five years. The project team will provide this professional learning free of charge. The project team will not offer any other compensation to teachers.

##### Professional learning for classroom teachers

Professional learning will assist teachers to implement three components: (i) quality PE and school sport, (ii) classroom movement breaks (known as ‘energizers’), and iii) physically active homework. To begin, an *iPLAY* mentor will facilitate one 2-h face-to-face workshop or two 1-h workshops on separate days at each school. After the initial workshop, teachers will complete eight online modules designed to reinforce and extend knowledge and skills gained in the initial workshop. During the workshop, mentors will encourage teachers to complete the online modules in small groups approximately once per month (e.g., at stage meetings). This collaborative approach is intended to foster development of an *iPLAY* community of practice within each school [36]. However, modules can also be completed independently.

At the end of the face-to-face workshop, each teacher will create an individualized learning plan. The learning plan will describe when each teacher intends to complete each of eight modules. The website/app will suggest to teachers that the learning plan accommodates at least one week between modules. This one-week interval will allow teachers time to implement and reflect on each teaching strategy. Upon completion of each module, the website/app will prompt teachers to reflect on their learning plan and adjust target dates, as required. Teachers will also have the ability to modify this learning plan at any time – i.e., without prompting. During the intervention, teachers will be prompted via a notification on their smartphone and/or an email when a new module is due for completion (according to each teacher’s self-selected, individualized learning plan).

Online learning activities will include brief instructional videos and engaging tasks that allow teachers to understand the rationale behind each teaching strategy [28]. Each module will be designed to take 30 min to complete, but teachers will be able to stop and start mid-module. Each module will include an action plan task in which teachers will set implementation goals for their PE and sport lessons. At the beginning of each online module, teachers will reflect on their progress towards goals set in the action plan from the previous module. In addition to the website, professional learning will also be available via a smartphone app on both iOS and Android platforms. In our recent professional learning trial [28], 109 of 110 NSW teachers owned a smartphone with one of these two operating systems. Thus, we expect most teachers in the proposed study will be able to access the app.

An *iPLAY* mentor will be assigned to each school and will observe one 30-min PE or sport lesson delivered by each consenting classroom teacher. Mentors will then meet individually with each teacher for 30 min to promote and guide self-reflection and provide feedback concerning the observed lesson. Feedback from mentors will be guided by an online observation checklist that prompts mentors to discuss the SAAFE (Supportive, Active Autonomous, Fair and Enjoyable) teaching principles [22], which are based on self-determination theory tenets [37]. During this conversation, the classroom teacher will answer reflective questions on the website/app.

Recently introduced regulations in NSW mandate that teachers engage in peer lesson observation. In *iPLAY*, teachers will be observed by one of their peers while they teach a 30-min PE or sport lesson. Afterwards, the pair will use a SAAFE checklist hosted on the project website/app as the basis for a 30-min peer discussion activity. As in the *iPLAY* mentoring session, classroom teachers will answer reflective questions on the website/app during the peer discussion activity.

The final training component for teachers will involve a 30-min small group discussion led by one of their school’s *iPLAY* leaders. During this meeting teachers will use the website/app to answer reflective questions regarding their implementation of *iPLAY* components. These meetings will likely take place during regularly scheduled ‘Stage Meetings’ involving teachers from (i) Early Stage 1 and Stage 1 – Kindergarten, Years 1 and 2, (ii) Stage 2 – Years 3 and 4 and (iii) Stage 3 – Years 5 and 6.

Teachers who join a school after the *iPLAY* intervention has started and/or miss the face-to-face workshop will be able to complete an online version of that component. They will complete all other aspects of the program as usual unless they join the school after the *iPLAY* intervention has finished and an *iPLAY* mentor is not available for the lesson observation component. In this instance, *iPLAY* leaders will be asked to facilitate this component.

##### Classroom teacher delivery

Support for classroom teachers’ implementation of the curricular components will include smartphone prompts, teaching resources, a class activity monitoring system and the mentoring described previously. The *iPLAY* smartphone app will provide reminders for teachers to implement strategies from their action plan. Teachers will be able to choose the interval for these reminders. The website and smartphone app will allow teachers to download resources (e.g., lesson plans, activity descriptions, and classroom movement break videos) that support intervention implementation. Also, when teachers set their action plan in each module, the web-based platform will identify resources that are specifically relevant to the skills/activities that the teacher has planned for the coming weeks. Links to these resources plus the action plan will be emailed to the teacher.

In the original *SCORES* intervention, teachers used Yamax digital pedometers (Yamax, Eagle Farm, Australia) and an Excel spreadsheet with an evidence-based algorithm [38, 39] to calculate the mean proportion of time their students spent being active during PE lessons. We have developed an activity tracking system that provides this information instantaneously to teachers at the end of a lesson. The system utilizes inexpensive pedometers ($20USD) (SmartLAB Move ANT+ pedometer, HMM, Dossenheim, Germany) that communicate wirelessly with a smartphone app. Each school will be provided with one activity tracking system which includes 25 pedometers, a smartphone pre-loaded with the app, and a carrying case that includes a charging station. Mentors will demonstrate the system to teachers in the school-based workshops and provide clarification as required when they observe each teacher’s lesson. An instructional video will form part of one of the online modules. A complete user manual will be available in the resource section of the website. In the action plans that teachers complete during online learning modules, they will be asked to indicate how many times they plan to use the physical activity monitoring system in their upcoming lessons. They will also have the option to set a goal for their students’ activity levels during these monitored lessons.

##### iPLAY leader training

We will work with school principals to recruit up to three *iPLAY* Leaders per school. These teachers will deliver the non-curricular components of the intervention (e.g., active playgrounds) and support other teachers with their implementation of the curricular components.

Each *iPLAY* leader will complete a series of four online learning modules (30 mins × 4 modules = 2 hours) designed to teach them how to implement the non-curricular components of the intervention (see Table 1 for details).

##### iPLAY leader delivery

Once all leaders in a school have completed the online training, the leaders will meet as a group with their school’s *iPLAY* mentor. The purpose of this 1-h meeting will be to set implementation goals for each non-curricular component and to determine the specifics of how leaders will support classroom teachers’ delivery of the curricular components (i.e., who will do what and when). The *iPLAY* leaders’ implementation plan for each school will be recorded on the website. As leaders make implementation progress in their schools, they will log this information, including reflections on facilitators and barriers.

In addition to recording their implementation of the non-curricular components on the website/app, leaders will be asked to meet with their school’s *iPLAY* mentor for one hour once per term to discuss progress and set new implementation goals. This meeting will also provide an opportunity for leaders and mentors to discuss classroom teachers’ implementation of the curricular components. Checklists to guide these meetings will be available on the website and mentors will be responsible for ensuring these are logged at the end of the meeting.

Finally, *iPLAY* leaders will facilitate at least one 30-min small group discussion session (~10 teachers/group) during which teachers in their school will reflect on their implementation of *iPLAY* components. Mentors will suggest to leaders that these meetings take place in the final term of the intervention.

#### Implementation timeline

**Fig. 3:**
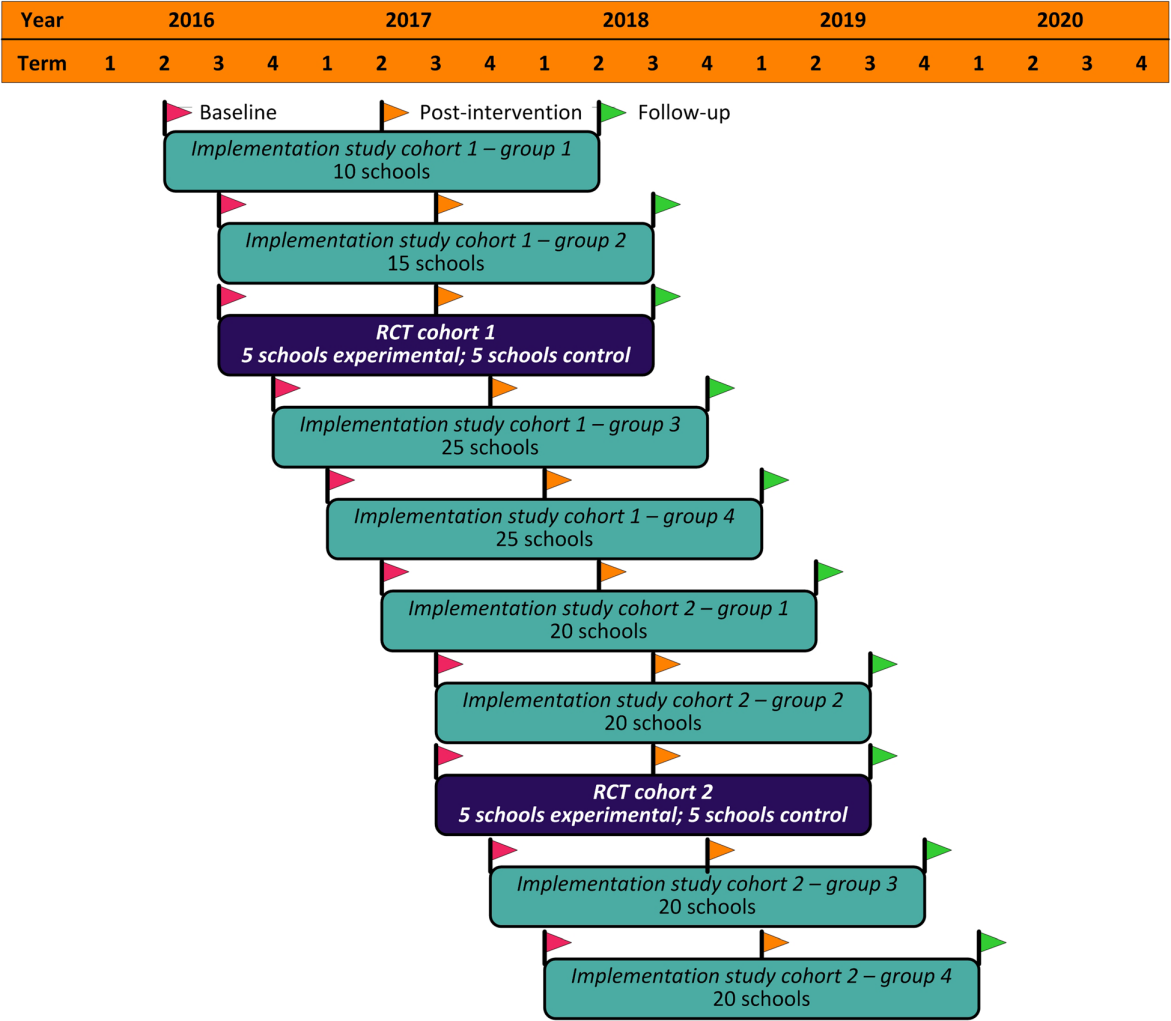
iPLAY Randomised Controlled Trial and Implementation Study Timelines

Within each cohort, the main *iPLAY* intervention will be delivered in four phases that roughly equate to 3.5 school terms (see Fig. 2), which is approximately 10 months. In the RCT, the five *iPLAY* intervention schools from Cohort 1 will begin the intervention starting in August 2016 (Term 3), while Cohort 2 is scheduled for June 2017 (Term 2). In the scale-up implementation study, the 80 schools in Cohort 1 will be divided into 4 groups that will begin the intervention on a rolling basis – Group 1 (June 2016 – Term 2), Group 2 (August 2016 – Term 3), Group 3 (November 2016 – Term 4) and Group 4 (March 2017 – Term 1). A similar roll-out is scheduled for Cohort 2, starting in June 2017. See Fig. 3 for details.

#### Ongoing implementation

At the end of the main intervention period (3.5 school terms = approximately 10 months), teachers will continue to have access to the *iPLAY* website and will have the ability to set action plans and access resources as often as they like. They can also re-visit online learning modules. Finally, *iPLAY* leaders in each school will have the option to lead up to 4 × 30 min *iPLAY* discussions with classroom teachers each year. These discussions will focus on *iPLAY* action planning and will include discussion of facilitators and barriers to implementation. Classroom teachers who participate in these discussions and complete a reflection task and an action plan via the website will earn up to an extra two BOSTES registered professional learning hours per year on top of the 14 h earned in the main *iPLAY* intervention.

### Cluster randomized controlled trial

We will conduct a cluster RCT with an allocation ratio of 1:1 (intervention : attention control) that conforms with CONSORT guidelines [40]. We will perform assessments at baseline, post-intervention (12 months after baseline) and maintenance (24 months after baseline).

#### Attention control Arm

Teachers in the 10 schools allocated to the attention control arm will be offered teacher professional learning designed to improve their delivery of the NSW Kindergarten–Year 6 Science and Technology curriculum. This program, known as *My Science*, has been shown to increase teacher confidence and student engagement in science [41]. Teachers who complete the *My Science* program will receive 10.5 h of BOSTES-registered teacher professional learning credit. They will also have the option to complete the *iPLAY* program at the end of the study, and earn an additional 14 h of registered professional learning credit.

The primary purpose of employing an attention control intervention is to limit principals’ and teachers’ disappointment at not receiving the *iPLAY* intervention, thereby increasing participation during data collection at the post-intervention and maintenance phases.

#### Participants

As noted previously, schools designated as ‘Schools for Specific Purposes’ will not be eligible for the cluster RCT. Schools that participated in the original *SCORES* efficacy study will also be excluded from the cluster RCT. All teachers in each school selected for the cluster RCT will be invited to participate in the intervention, but only students in Years 3 and 4 will complete outcome assessments.

#### Procedure

Principals and teachers will provide written informed consent to participate in the cluster RCT. Students will provide assent and parents will provide written informed consent for their child to participate. Trained research assistants will collect all student level outcomes in the cluster RCT. These data collectors will not be informed of schools’ allocation to the intervention or control condition; however, due to the use of social marketing within *iPLAY* schools (e.g., posters), our ability to meaningfully blind these researchers is significantly diminished. Despite this limitation, the potential risk of bias for many measures in this study is low (e.g., objective measures of physical activity) and statisticians will be blinded to each school’s allocation.

### Primary outcome measure – entire RCT sample

Cardiorespiratory fitness will be assessed using the 20 m multistage fitness test [42], which has demonstrated strong validity in studies worldwide [43] and is considered to be the most appropriate field-based measure [44]. We will measure cardiorespiratory fitness for all physically able children in the cluster RCT.

### Secondary outcome measures

#### Student level measures – entire RCT sample

##### Student physical activity (objective measure)

We will measure students’ physical activity behavior over a period of eight days using GENEActiv accelerometers (Activinsights, Cambridge, United Kingdom) worn on the non-dominant wrist. GENEActiv accelerometers are valid for children [45], and wrist-based accelerometry may be more acceptable for children compared with hip-worn monitors, resulting in greater compliance and reducing missing data [46]. Data will be reduced using evidenced-based, best-practice procedures at the time of analysis. At present, this involves using the Euclidean norm minus one (ENMO) method to apply cut-points [45] to the data, providing estimates of time in different intensities of activity (e.g., moderate vs. vigorous). Accelerometry data will be used to examine: (i) within school activity, (ii) recess and lunch activity, (iii) after-school activity, (iv) weekend activity and (v) total activity.

##### Anthropometry

We will measure all students’ height and weight, using stadiometers (Surgical and Medical Products No. 26SM, Medtone Education Supplies, Melbourne, Australia) and digital scales (UC-321, A&D Company LTD, Tokyo, Japan), respectively. We will then calculate body mass index (BMI) and BMI z-scores using the Centers for Disease Control and Prevention methodology [47].

##### Student characteristics

Students will self-report their sex and date of birth. They will also indicate the country in which they were born and the language they speak at home. We will use this information to categorize students into one of seven ethnic backgrounds (English, European, Middle Eastern, Asian, African, South Pacific or ‘other’), based on the Australian Bureau of Statistics’ Standard Classification of Languages [48]. We will also ask students to indicate if they are of Indigenous origin (i.e., Aboriginal or Torres Strait Islander). We will assess student-level socioeconomic status through the child’s self-reported home suburb, children’s perception of the number of books in their home (as measured in Trends in International Mathematics and Science Study) [49], and a single-item question on perceived socioeconomic status [50].

##### Student physical activity (self-report)

We will measure students’ activity behaviors using single item measures of (i) typical physical activity participation [51], (ii) physical activity participation last week [51], (iii) organized sport participation in the past year with team and individual sports measured separately [52] and (iv) active commuting to school [52].

##### Teachers’ interpersonal style during PE and school sport

We will use a 4-item scale to assess students’ perceptions of their teacher’s support of students’ psychological needs. This will involve two items from an adapted version of the Teacher as Social Context questionnaire [53], one item adapted from the Health Care Climate Questionnaire [54] and one item from the Controlling Teacher Scale [55].

##### Student behavior, affect and cognition during PE and school sport

We will assess effort through three items, including two items from the Student Engagement in School questionnaire [56] and one item from the effort subscale of the Intrinsic Motivation Inventory [57]. *Enjoyment* will be assessed using three items adapted to PE and school sport from the Student Engagement in School questionnaire. Three items will be used to assess students’ concentration in the lessons [58]. Three items from the *Use of Strategies* subscale of the Cognitive Processes Questionnaire in Physical Education [59] will measure strategies students employ when learning in PE and school sport.

##### Subjective well-being

We will measure students’ perceived well-being using 10 items from the WHO’s Health Behavior in School-aged Children questionnaire [50].

##### Academic achievement

We will work with NSW BOSTES to obtain students’ Year 3 and 5 NAPLAN numeracy and literacy standardized test scores [60].

### Student level measures – sub-sample

Within each school, we will randomly select one class to form a sub-sample. We expect 18 students per class to volunteer; therefore, the subsample will include approximately 360 children. Children in the sub-sample will complete the following measures in addition to the previously described measures.

#### Fundamental movement skill competency

Students’ fundamental movement skill competence will be measured using three object-control skills from the Test of Gross Motor Development-2 [61]. From the 12 skills available, we selected the overarm throw, catch, and kick due to their transferability into a variety of different sports that are popular among Australian children. Moreover, object control skills are most strongly associated with physical activity levels in comparison to locomotor and stability skills [62, 63].

#### Cognitive control

We will measure children’s working memory and inhibition using a modified AX-Continuous Performance Task (AX-CPT) [64]. The tests will be administered by trained research assistants and completed by participants using a computer. The AX-CPT requires participants to correctly respond to target trials that occur when the letter ‘X’ (correct-probe) is immediately preceded by the letter’A’ (correct-cue). Non-target trials occur when probes are letters other than X (collectively referred to as’Y’) and/or cues are letters other than A (referred to collectively as’B’). Thus, participants encounter four types of trials: AX, AY, BX, and BY [65].

### Principal level measures – entire sample (online questionnaires)

#### Principal characteristics

Principals will self-report their demographic information (age, sex, ethnicity, and number of years teaching). Additionally, we will ask principals to declare if they have ever been accredited as a specialist PE teacher, and to self-report their physical activity [51] and sport participation [52].

#### School characteristics

Principals will complete items measuring the number of classes, number of students per class, number of employed teaching staff within their schools, number of PE specialist teachers and bell times for the school (e.g., school start, recess, lunch, and school end times).

#### School physical activity

We will assess principals’ perceptions of facilities, equipment, time allocation, and support for physical education in their school using 13 items from the NSW School Physical Activity and Nutrition Survey [66]. A single-item measure will be used to determine if schools currently receive ‘*Sporting Schools*’ funding for external providers to run sport programs within the school.

### Teacher level measures– entire sample (online questionnaires)

#### Teacher characteristics

As with principals, teachers will self-report their demographic information (date of birth, sex, ethnicity, and number of years teaching). We will also ask teachers to report the stage they are currently teaching, and their current level of BOSTES accreditation. Additionally, we will ask teachers to declare if they have ever been accredited as a specialist PE teacher, and to self-report their physical activity [51] and sport participation [52].

#### Teacher confidence

We will assess teacher confidence in teaching PE and school sport, as well as other key learning areas (e.g., English, Mathematics, Science and Technology), by adapting a measure of non-specialist primary teachers’ confidence to teach PE [67].

#### Student conduct

A single item measure will be used to assess teachers’ perceptions of their students’ behavior [56].

#### Perceived student engagement

We will measure teachers’ perception of their students’ engagement in PE and school sport lessons, as well as other key learning areas (e.g., English, Mathematics, Science and Technology) using an adapted version of the Student Engagement in School Questionnaire [56].

#### Internet self-efficacy

An eight-item Internet self-efficacy scale will be used to assess teachers’ beliefs in their ability to utilize internet tools [68].

#### Job satisfaction, burnout and absenteeism

Single-item measure of overall job satisfaction [69] and burnout [70] will be used. Additionally, we will seek permission from teachers to collect from their principal the number of days absent from work due to illness.

##### Statistical analyses and sample size

We will test for between-arm differences in changes in student outcomes using linear mixed models with standard errors corrected for clustering. We will analyze data according to intention to treat principles (main analyses) and per-protocol principles (sensitivity analyses). We conducted a power analysis using procedures appropriate for complex nested designs [71]. In this analysis the effect size for between-arm differences in cardiorespiratory fitness (primary outcome) was conservatively set to .35 (note: effect in our efficacy trial was .54) with intraclass correlations based on our efficacy trial [21] (class = .09, school = .01). Analysis indicated that 1080 students from 60 classes in 20 schools (i.e., 3 classes per school) would provide power of .91.

We will explore potential moderators of intervention effects including children’s age, sex, ethnicity, weight status and SES, as well as baseline levels of cardio-respiratory fitness, physical activity and fundamental movement skill competence. As with the main analyses, we will employ a mixed modeling approach to explore moderation hypotheses by including appropriate interaction terms in the regression models. The trial is not powered to detect interactions; thus, we will employ a significance level of *p* < 0.1 to explore potential moderators. We will explore significant interaction terms by testing sub-groups differences on the primary outcome and selected secondary outcomes. We will also explore potential moderating effects of principal and teacher characteristics (e.g., specialist PE accreditation) on student outcomes.

Per protocol analyses will investigate the influence of *iPLAY* leaders’ and other teachers’ adoption and implementation of the intervention on student outcomes. Adoption will focus on the proportion of intervention training components completed (e.g., workshops attended and online tasks completed), while implementation will evaluate leaders’ and teachers’ utilization of strategies in their schools (as per Table 1).

Linear mixed models will be also used to examine potential mediating processes. For example, in our efficacy study we found that changes in fundamental movement skills mediated the effect of the intervention on children’s physical activity and cardiorespiratory fitness. Mediating effects will be estimated using a cluster-bootstrapped based product-of-coefficients test that is appropriate for cluster RCTs.

##### Economic evaluation

We will conduct an economic evaluation to determine if *iPLAY* represents ‘value-for-money’ measured incrementally against the attention control arm. This allocative efficiency focus will determine whether the cost of the intervention is justified by the benefits derived from it, measured against usual practice. Costs in each arm of the trial will be estimated from a societal perspective using detailed pathway analysis to identify resource use, measurement and valuation processes for the reference year 2018. The incremental differences in costs will be combined with the behavioral and biophysical outcomes observed in the trial to produce a range of incremental cost effectiveness ratios. In addition to a ‘trial-based evaluation’ (costs and outcomes exactly as per the trial), depending on the outcomes, a modelled economic evaluation with the extended time horizon may be undertaken to further translate the benefits observed in the trial into final health benefits, assessed as disability-adjusted life years (DALYs) averted. The modelled economic evaluation will simulate the impact of increased physical activity and movement skill competency on overall well-being over the lifetime of the cohort compared with usual practice. A Markov model [72] consisting of health states associated with different levels of physical activities/movement skill competency will be used to accrue costs and benefits over the time horizon. The long-term improved outcome may translate into cost savings which offset the increased cost associated with the implementation of *iPLAY* project. Simulation-modelling using the @RISK software package will be used to calculate 95 % uncertainty intervals around the epidemiological probabilities and cost estimates.

### Scale-up implementation evaluation

Running alongside the cluster RCT will be a scale-up implementation study. This evaluation will be a multiple cohort design, with all schools receiving the intervention. Measurement will be guided by the RE-AIM framework [73] and will occur at baseline, 12 and 24 months for each cohort.

#### Participants and procedures

Participants will include principals, teachers and students at government-funded primary schools in NSW. There will be no exclusion criteria for principals or teachers within these schools. To be included in the study at least 50 % of Stage 2 (Years 3 and 4) teachers must be willing to participate in the program, at least one staff member must be willing to be an *iPLAY* leader, and the principal must provide consent for the program to run in the school. All students who are enrolled in Years 3 or 4 (Stage 2) at baseline and who are able to participate in physical activity will be eligible for the study, except for students enrolled in ‘Schools for Specific Purposes’ (i.e., for students who require intensive levels of support). In these schools, teachers will be eligible to participate in the study, but students will not be asked to complete outcome assessments.

Principals and teachers will provide written informed consent to participate in the scale-up implementation study. Passive consent procedures will be used regarding student participation; newsletters will be sent home and will ask parents to indicate if they do not wish their child to participate in the study.

#### Measures

##### Reach

We will examine the extent to which participating schools are representative of the NSW population, in terms of school size, SES, and location. Once a school is recruited into the study we will employ a questionnaire to ask the principal to identify the “single most important reason for your decision to participate”. At the end of recruitment, we will purposively sample 100 schools (according to size, SES, and location) that do not volunteer and follow-up by telephone to determine reasons for non-participation.

##### Effectiveness

We will conduct a reduced examination of effectiveness in the scale-up implementation study compared with the cluster RCT. Assessments will include all questionnaires and standaridized tests of numeracy and literacy from the cluster RCT. Other measures (e.g., 20 m multistage fitness test, accelerometers, fundamental movement skills, and cognitive control) will not be obtained in the scale-up implementation study.

Principals and teachers will complete online questionnaires. Classroom teachers will also administer an online questionnaire to their students to complete self-report measures. Questionnaires will be administered to principals, teachers, and students at baseline, post-intervention (12 months) and maintenance (24 months).

##### Adoption

We will examine the proportion of schools from the NSW population that volunteer and participate in the program. We will assess teacher level adoption by examining the proportion of teachers who complete each aspect of the training, including attendance at face-to-face workshops and completion of online components, as well as participation in mentor meetings, peer observations and small group discussions.

##### Implementation

We will monitor implementation as per Table 1 (curricular and non-curricular components).

##### Maintenance

Using the procedure described above, we will re-examine effectiveness and implementation 24 months after baseline. To further understand barriers and facilitators to implementation, we will conduct semi-structured interviews with purposively selected principals (*n* = 15), teachers (*n* = 15) and students (*n* = 15). Sampling will ensure that interview participants are drawn from schools in which the intervention had strong effects, weak effects and no effects. Thematic analysis of transcripts will indicate ways to improve implementation prior to further dissemination.

#### Statistical analyses

The scale-up implementation study will be assessed with a focus on descriptive statistics concerning reach, adoption, implementation, and maintenance. We will also use linear regression to explore the impact of school and community characteristics on program reach. We will use linear mixed model analysis to examine changes in outcome variables from baseline to 12-months and 24-months (i.e., effectiveness). These effects will be estimated for the entire sample as well as in key sub-populations (e.g., across teacher sex, school average SES, teachers with high vs low internet self-efficacy). Where possible, we will compare *iPLAY* schools with expected values within the population (e.g., NAPLAN scores in similar schools, physical activity participation) from other data sources such as NSW School Physical Activity and Nutrition Survey [66]. We will also compare outcomes at 12 months (post-intervention) for Cohort 1 with baseline levels for Cohort 2, taking advantage of the natural experiment that is inherent in the design of the study.

#### Economic evaluation

The research question for economic evaluation of the scale-up implementation study will be to assess, from a societal perspective, the cost-outcome of scaling up the *iPLAY* project (rollout and implementation to 160 schools) in primary schools within NSW to assess intervention affordability and sustainability.

The economic analysis will be a cost-outcome description as the one-arm design of the scale-up implementation study does not include a control arm (which is necessary for determination of comparative cost-effectiveness). The primary economic analysis will comprise three components: a cost analysis; an outcome analysis and the relationship between cost and outcomes for the intervention. Costing of the intervention using opportunity cost principles will involve the following steps:

- Identification of costs to be included, using ‘pathway analysis’, where activities in all stages of the roll out of the *iPLAY* project are fully specified; A societal perspective and steady state operation of the intervention will be assumed (up and running to its full effectiveness potential). Costs will largely relate to the time costs of specialist mentors, leaders, classroom teachers, and school principals (using opportunity cost principles). Any administrative resources used at the program management level also will be identified and included, although research-driven activities will be separated from the activities that would be carried out should the program be adopted by primary schools;
- Measurement of the resources consumed in natural units (number of hours spent by specialist mentors/leaders within school/principals to deliver the intervention, number and length of school visits, etc.);
- Valuation of these resources in monetary units (using 2018 as the reference year).

In addition, variations in delivery costs of the *iPLAY* intervention between participating schools will be identified in order to determine any factors that may impact on the roll out of this program throughout NSW primary schools and its adoption in other jurisdictions.

The economic outcomes for the scale-up implementation study will be presented as total costs, average costs per child and per school, separately from the intervention and maintenance periods. The relationship between costs and outcomes will be reported as average cost per outcome.

## Discussion

The purpose of this study is to evaluate the extent to which an existing, efficacious physical activity intervention can be scaled-up and disseminated widely using online learning methods alongside face-to-face implementation support. A web-based delivery system is attractive as it may support scaling-up and sustainability. However, little, if any, evidence exists regarding the effectiveness of comprehensive primary school-based physical activity interventions delivered using online methods. Using two concurrent studies, and guided by the RE-AIM framework, our project will help provide evidence on the effectiveness and cost-effectiveness of teacher professional learning delivered largely via the Internet to address the issue of physical inactivity among primary school-aged children.

## Abbreviations

BMI, body mass index; BOSTES, Board of Studies, Teaching and Educational Standards; iPLAY, Internet-based Professional Learning to help teacher support Activity in Youth; MVPA, moderate-to-vigorous physical activity; NAPLAN, National Assessment Program – Numeracy and Literacy; PE, Physical Education; RCT, randomized controlled trial; RE-AIM, Reach Effectiveness – Adoption Implementation Maintenance; SCORES, Supporting Children’s Outcomes using Rewards, Exercise and Skills; SES, socioeconomic status; WHO, World Health Organization
