# Erratum to: Scaling-up an efficacious school-based physical activity intervention: Study protocol for the ‘Internet-based Professional Learning to help teachers support Activity in Youth’ (iPLAY) cluster randomized controlled trial and scale-up implementation evaluation

**DOI:** 10.1186/s12889-016-3724-3

**Published:** 2016-09-30

**Authors:** Chris Lonsdale, Taren Sanders, Kristen E. Cohen, Philip Parker, Michael Noetel, Tim Hartwig, Diego Vasconcellos, Morwenna Kirwan, Philip Morgan, Jo Salmon, Marj Moodie, Heather McKay, Andrew Bennie, Ron Plotnikoff, Renata L. Cinelli, David Greene, Louisa R. Peralta, Dylan P. Cliff, Gregory S. Kolt, Jennifer M. Gore, Lan Gao, David R. Lubans

**Affiliations:** 1 Institute for Positive Psychology and Education, Australian Catholic University, Edward Clancy Building 167-169 Albert St, Strathfield, NSW 2135 Australia; 2 Priority Research Centre for Physical Activity and Nutrition, School of Education, University of Newcastle, Callaghan, NSW 2308 Australia; 3 Institute for Positive Psychology and Education and School of Exercise Science, Australian Catholic University, Edward Clancy Building 167-169 Albert St, Strathfield, NSW 2135 Australia; 4 School of Exercise Science, Australian Catholic University, Edward Clancy Building 167-169 Albert St, Strathfield, NSW 2135 Australia; 5 Physical Activity Research Group, School of Human Health and Social Sciences, Central Queensland University, Building 18, Yaamba Road, Rockhampton, QLD 4702 Australia; 6 Institute for Physical Activity and Nutrition (IPAN), School of Exercise and Nutrition Sciences, Deakin University, Geelong, Australia; 7 Deakin Health Economics, Centre for Population Health Research, Faculty of Health, Deakin University, Geelong, VIC Australia; 8 Center for Hip Health and Mobility, University of British Columbia, 7/F, 2635 Laurel Street, Vancouver, BC V5Z 1 M9 Canada; 9 School of Science and Health, Western Sydney University, Locked Bag 1797, Penrith, NSW 2751 Australia; 10 School of Education, Australian Catholic University, 250 Victoria Parade East, Melbourne, VIC 3002 Australia; 11 Faculty of Education and Social Work, University of Sydney, Sydney, NSW 2006 Australia; 12 Early Start Research Institute, School of Education, University of Wollongong, Wollongong, NSW 2522 Australia; 13 Teachers and Teaching Research Centre, School of Education, University of Newcastle, Callaghan, NSW 2308 Australia

## Erratum

After publication of the original article [1] it was brought to our attention that author Diego Vasconcellos was incorrectly included as Diego Vasoncellos. The correct spelling of the name is included in the author list of this erratum and updated in the original article.

## References

[1] Lonsdale, et al. Scaling-up an efficacious school-based physical activity intervention: Study protocol for the ‘Internet-based Professional Learning to help teachers support Activity in Youth’ (iPLAY) cluster randomized controlled trial and scale-up implementation evaluation. BMC Public Health. 2016;16:873. doi:10.1186/s12889-016-3243-2. 10.1186/s12889-016-3243-2 PMC4997792 27557641

